# Hypophosphatemia Correction Reduces ICANS Incidence and Duration in CAR T-cell Therapy: A Pooled Clinical Trial Analysis

**DOI:** 10.1158/2767-9764.CRC-24-0250

**Published:** 2024-10-03

**Authors:** Jack Pengfei Tang, Penelope Lafeuille, Alexandru Socolov, Sheila S. Diamond, Jacob Aptekar, Theodore B. Moore, Esther H. Nie, Mark R. Hanudel, Theodore S. Nowicki

**Affiliations:** 1 Division of Pediatric Hematology-Oncology, Department of Pediatrics, University of California Los Angeles, Los Angeles, California.; 2 Medidata, a Dassault Systèmes Company, New York, New York.; 3 Division of Neuroimmunology, Department of Neurology and Neurological Sciences, Stanford University, Palo Alto, California.; 4 Division of Pediatric Nephrology, Department of Pediatrics, University of California Los Angeles, Los Angeles, California.; 5 Department of Microbiology, Immunology, and Molecular Genetics, University of California Los Angeles, Los Angeles, California.; 6 Jonsson Comprehensive Cancer Center, University of California Los Angeles, Los Angeles, California.; 7 Eli and Edythe Broad Center for Regenerative Medicine and Stem Cell Research, University of California Los Angeles, Los Angeles, California.; 8 Molecular Biology Institute, University of California Los Angeles, Los Angeles, California.

## Abstract

A common complication of chimeric antigen receptor (CAR) T-cell therapy is immune effector cell–associated neurotoxicity syndrome (ICANS), which presents with encephalopathy, aphasia, inattention, somnolence, seizures, weakness, or cerebral edema. Despite its significant morbidity, there are currently no effective targeted treatments. Given the clinical similarities between ICANS and the neurological manifestations of acute hypophosphatemia, we retrospectively reviewed 499 patients treated with CD19-targeted CAR T-cell therapy across multiple clinical trials between 2015 and 2020. In addition to clinical toxicities experienced by the patients, we also interrogated the impact of serum electrolyte data and repletion of corresponding electrolyte deficiencies with ICANS incidence, severity, and duration. Hypophosphatemia was a common occurrence in CAR T-cell recipients and the only electrolyte derangement associated with a significantly higher cumulative incidence of ICANS. Moreover, phosphorus repletion in patients with hypophosphatemia was associated with significantly decreased ICANS incidence and duration. Hypophosphatemia was uniquely associated with encephalopathy neurological adverse events, which also showed the strongest positive correlation with both ICANS and cytokine release syndrome severity. These findings suggest that serum phosphorus could be a reliable biomarker for ICANS, and expeditious, goal-directed phosphorus repletion in response to serum hypophosphatemia could be a safe, inexpensive, and widely available intervention for such patients.

**Significance:**

Herein we show that phosphorus repletion in patients with hypophosphatemia receiving anti-CD19 chimeric antigen receptor T-cell therapeutics was associated with significantly decreased immune effector cell–associated neurotoxicity syndrome (ICANS) incidence and symptom duration. Given the significant morbidity associated with ICANS and lack of targeted interventions, hypophosphatemia may serve as both a useful biomarker and an inexpensive intervention for ICANS.

## Introduction

Chimeric antigen receptor (CAR) T-cell therapy has revolutionized the treatment of hematologic malignancies, including B-cell leukemias and lymphomas (1). Despite their clinical success, CAR T-cell therapies are associated with two significant toxicities: cytokine release syndrome (CRS) and immune effector cell–associated neurotoxicity syndrome (ICANS), both of which require stringent inpatient monitoring and often result in prolonged hospitalization and escalation to intensive care settings. ICANS, which generally occurs in patients who have experienced prior or concomitant CRS, manifests as confusion, inattention, word-finding difficulty, aphasia, impaired motor skills, and/or somnolence. In severe cases, ICANS symptoms can include neurological weakness, seizures, cerebral edema, and coma (2, 3). Although CRS can be managed using targeted agents that do not inhibit therapeutic efficacy of the CAR-T cells, such as the IL6 receptor antagonist tocilizumab, the mainstay of ICANS treatment is supportive care and nonspecific immunosuppression with corticosteroids (4). Even with corticosteroid use, there is no consensus regarding optimal dosage, duration, or timing, with studies exploring prophylactic use showing continued high ICANS incidence (5). Additionally, there are concerns that nonspecific immunosuppression with steroids may impact the antitumor activity of CAR T-cell therapy (6). Therefore, there is an unmet need for reliable biomarkers of ICANS, as well as early and specifically targeted interventions for its treatment.

Our group and others have previously reported the association between hypophosphatemia and ICANS. This is of interest given the striking similarities between ICANS and the neurological manifestations of acute hypophosphatemia, including encephalopathy, confusion, altered mental status, seizure, and coma (7, 8). Hypophosphatemia can occur because of drastic and sudden increases in a patient’s metabolic demand, such as in sepsis or refeeding syndrome, as the increased intracellular demand for phosphorylated metabolic intermediates leads to rapid redistribution of extracellular phosphorus into cells (9, 10). In CAR T-cell recipients, we have previously shown that hypophosphatemia may be driven in part by the increased antigen-dependent metabolic activity of the CAR T-cells and the increased metabolic demand that occurs in the setting of increased systemic inflammation due to CRS (7).

Here, we examined further the relationship between electrolyte derangements and ICANS in a large pooled clinical cohort of patients receiving CD19-targeted CAR T-cell therapies. We also explored the impact of different electrolyte repletions on ICANS incidence and symptom duration, as well as the association between electrolyte derangements and various categories of neurologic adverse events observed in CAR T-cell recipients. We report herein the association between hypophosphatemia and ICANS incidence, as well as our findings that phosphorus repletion in the setting of hypophosphatemia significantly decreases ICANS incidence and symptom duration, highlighting the potential of hypophosphatemia correction as a targeted intervention in ICANS management.

## Materials and Methods

### Retrospective analysis of CD19-targeted CAR T-cell clinical cohort

We retrospectively reviewed the clinical details of 499 deidentified adult patients with acute lymphoblastic leukemia (ALL) or non–Hodgkin lymphoma (NHL) treated with CD19-targeted CAR T-cell therapy with CD28 costimu–latory domains across multiple clinical trials conducted between 2015 and 2020. These data were made available through the Medidata Clinical Cloud, which stores aggregated and anonymized study data from trials run on Medidata’s platform. Medidata harmonizes and standardizes the anonymized data using industry-compliant Common Data Models, such as Study Data Tabulation Model and Analysis Data Model. In accordance with 45 CFR §46, this study’s cohort was exempt from institutional review board review and the need for written informed consent because the unit of analysis was the trial and the analytical data contained no identifying information regarding trial sponsors, agents, or accrual sites. Clinical details included demographic information (age and sex), clinical diagnosis, adverse reactions, treatment response, and common laboratory values such as serum electrolytes (phosphate, potassium, magnesium, and calcium), inflammatory markers (C-reactive protein), creatinine, and serum albumin. CRS and ICANS severity were graded from 1 to 4 based on the 2019 American Society for Transplantation and Cellular Therapy (ASTCT) guidelines (3). These were manually assigned by a neuroimmunologist at the time of review based on reported neurological adverse events (NAEs). Treatment response (complete response, partial response, stable disease, and progressive disease) was obtained directly from case report forms from the clinical trials. We defined hypophosphatemia as serum phosphorus level <2.5 mg/dL, hypokalemia as serum potassium level <3.0 mmol/L, hypomagnesemia as serum magnesium level <1.3 mg/dL, and hypocalcemia as corrected serum calcium level <8.0 mg/dL. Serum calcium was corrected based on serum albumin levels. Estimated glomerular filtration rate (eGFR) was calculated using the Chronic Kidney Disease Epidemiology Collaboration (CKD-EPI) formula (11). The incidence and severity of acute kidney injury (AKI) was determined based on changes in serum creatinine (Scr) after CAR T-cell infusion throughout the hospital course according to the Kidney Disease Improving Global Outcomes criteria (12). A subset of patients received preemptive treatment with tocilizumab (8 mg/kg) once immediately between day −7 and day +28 from CAR T-cell infusion but before any incidence of CRS and/or ICANS. Baseline tumor burden for patients (preinfusion) was defined as area (cm^2^) for patients with NHL and peripheral blast:leukocyte ratio for patients with ALL.

### Electrolyte repletion analyses

To examine the effects of targeted electrolyte repletion, we first stratified patients based on the presence or absence of a given electrolyte derangement following CAR T-cell infusion based on the definitions listed above. Patients were subsequently stratified based on whether or not they received any repletion with the corresponding deficient electrolyte between the time of CAR T-cell infusion (day 0) until day 14 or onset of ICANS symptoms. Fisher exact test was used to compare the incidence of ICANS between these groups, and unpaired *t* test was used to compare the duration of ICANS symptoms.

### NAEs

To explore the association between ICANS grade, CRS grade, and various electrolyte derangements with specific neurological symptoms, we first mapped all individual NAEs recorded during the clinical trial to the MedDRA dictionary (13). Next, we grouped the mapped NAEs by High Level Group Term (MedDRA definition). The NAE groups were reviewed with a neuroimmunologist for clinical relevance. A full breakdown of consolidated NAE terms can be found in Supplementary Table S1. Then, for each patient, we calculated the Spearman rank correlation between the patient’s maximum Common Terminology Criteria for Adverse Events grade for each NAE term and the following covariates: ICANS grade, CRS grade, hypophosphatemia, hypokalemia, hypomagnesemia, and hypocalcemia. The rank correlations were then plotted as a bar graph grouped by NAE terms.

### Statistical analysis

Patients were grouped by ICANS status. Baseline demographic characteristics were reported as median values for continuous variables and as count and percentages for categorical variables. Fisher exact test was performed for categorical variables, and the Mann–Whitney test or unpaired *t* test was performed for continuous variables. Time-to-event data were summarized using the Kaplan–Meier method, wherein patients were stratified based on the presence or absence of electrolyte deficiencies (hypophosphatemia, hypokalemia, hypomagnesemia, and hypocalcemia, as defined by the above thresholds), and the time to ICANS occurrence was plotted. The log-rank test was then performed to compare groups. For all statistical investigation, an alpha/*P* value of <0.05 was used as the cut-off for significance for two-sided statistical testing. Graphing and statistical analyses were performed using GraphPad Prism V.10 (RRID:SCR_002798) or Python V3.12 (RRID:SCR_024202).

### Data availability

The data generated in this study are available upon request from the corresponding author.

## Results

### Patient characteristics

A total of 499 patients (*n* = 336 male, *n* = 163 female) with a median age of 58 years treated with CD19-directed CAR T-cell therapy for ALL (*n* = 134) or NHL (*n* = 365) across clinical trials conducted between 2015 and 2020 were included in this study. In this cohort, 55.5% of patients developed ICANS. CRS was observed in 88.8% of patients, including in 95.7% of patients who developed ICANS compared with 80.2% of patients without ICANS (*P* < 0.0001). Patient age, sex, disease indication, and treatment response were not significantly associated with the development of ICANS (Table 1).

**Table 1:** Demographics, outcomes, and selected laboratory values of patients treated with anti-CD19 CAR T-cell therapy stratified by ICANS status

	No ICANS (*n* = 222)	ICANS (*n* = 277)	*P* value
Age (median, years)	58	57	0.2892
Sex			0.7009
F	70	93	
M	152	184	
Diagnosis			0.6126
ALL	57	77	
NHL	165	200	
CRS	178 (80.2%)	265 (95.7%)	**4.86E-8**
Electrolyte disturbance			
Hypophosphatemia	134 (60.4%)	204 (73.6%)	**0.0020**
Hypokalemia	14 (6.3%)	16 (5.8%)	0.8510
Hypomagnesemia	6 (2.7%)	13 (4.7%)	0.3472
Hypocalcemia	20 (9.0%)	29 (10.5%)	0.6510
Nadir phosphorus (median, mg/dL)	2.2	2.0	**0.0072**
Nadir potassium (median, mmol/L)	3.5	3.4	**0.0346**
Nadir magnesium (median, mg/dL)	1.7	1.7	**0.0031**
Nadir calcium corrected (median, mg/dL)	8.6	8.6	0.4521
Baseline creatinine (median, mg/dL)	0.79	0.81	0.2843
Baseline eGFR (median, mL/minutes/1.73 m^2^)	95.0	93.0	0.6432
Peak C-reactive protein (median, mg/dL)	101.2	112.0	0.2354
AKI	24 (10.8%)	60 (21.7%)	**0.0016**
Stage 1	19 (8.6%)	38 (13.7%)	
Stage 2	0	11 (4.0%)	
Stage 3	5 (2.3%)	11 (4.0%)	
Treatment response^a^			0.2152
CR	131 (66.8%)	181 (72.4%)	
PR	33 (16.8%)	39 (15.6%)	
SD	18 (9.2%)	9 (3.6%)	
PD	14 (7.1%)	21 (8.4%)	

Abbreviations: CR, complete response PR, partial response; PD, progressive disease; SD, stable disease.

Values presented as median or count (percent).

*P* values <0.5 are bolded.

a Responders defined as CR + PR; nonresponders defined as SD + PD.

### Hypophosphatemia is associated with ICANS incidence in anti-CD19 CAR T-cell recipients

Following CAR T-cell infusion, a majority of patients developed hypophosphatemia (67.7%), whereas hypokalemia (6.0%), hypomagnesemia (3.8%), and hypocalcemia (9.8%) were experienced with far less frequency, and only hypophosphatemia was significantly associated with ICANS (*P* = 0.0020; Table 1). The median nadir serum phosphorus concentration in this cohort was in the hypophosphatemic range and was significantly lower in patients who developed ICANS than in those who did not (2.0 mg/dL vs. 2.2 mg/dL, *P* = 0.0072; Supplementary Fig. S1A). Median nadir serum potassium levels were also lower in patients who developed ICANS (3.4 mmol/L vs. 3.5 mmol/L, *P* = 0.0346; Supplementary Fig. S1B), and whereas nadir serum magnesium levels were statistically different between ICANS and non-ICANS groups, this was due to a difference in distribution, rather than magnitude (Supplementary Fig. S1C). No significant difference was observed in the median nadir values for corrected serum calcium levels (Supplementary Fig. S1D). Range and interquartile range of nadir values of electrolytes stratified by ICANS status are summarized in Supplementary Table S2. Temporally, we observed a trend of decreasing serum phosphorus levels following CAR T-cell infusion, with a greater magnitude of decline in patients who developed ICANS (Supplementary Fig. S2A), in contrast to the other electrolytes interrogated (Supplementary Fig. S2B–S2D). Moreover, the median time to nadir serum phosphorus was coincident with the median onset of ICANS symptoms on post-infusion day 6 (Supplementary Fig. S3A and S3B). Furthermore, our data showed that there was a significant association of hypophosphatemia with ICANS by grade (*P* < 0.01, one-way ANOVA; Supplementary Fig. S4).

For patients with CRS, we observed no significant difference in the incidence of hypophosphatemia between those patients with only CRS or patients with CRS and ICANS (Supplementary Table S3). Furthermore, we analyzed those subjects who did or did not receive prophylactic tocilizumab prior to CAR-T infusion (Supplementary Table S4). In the subjects who received prophylactic tocilizumab (*n* = 134), compared with those who did not receive prophylactic tocilizumab (*n* = 365), the rate of CRS is lower (86.6% vs. 92.3%, *P* = 0.055; Fisher exact test) and the rate of hypophosphatemia is lower (57.5% vs. 71.5%, *P* = 0.0035; Fisher exact test), but we observed no impact on the incidence of ICANS (58.2% vs. 54.5%, *P* = 0.48; Fisher exact test). However, within both groups, we observed a significant association between hypophosphatemia and ICANS incidence, as well as hypophosphatemia and CRS incidence. We found no significant difference in the pre-CAR-T infusion tumor burden of patients with NHL and the incidence of hypophosphatemia or ICANS (Supplementary Fig. S5A and S5B). For the patients with ALL, there was a significant association with reduced incidence of ICANS and a weak association with reduced incidence of hypophosphatemia (*P* = 0.07; Supplementary Fig. S5C and S5D).

### Serum hypophosphatemia is associated with a higher cumulative incidence of ICANS

**Figure 1:**
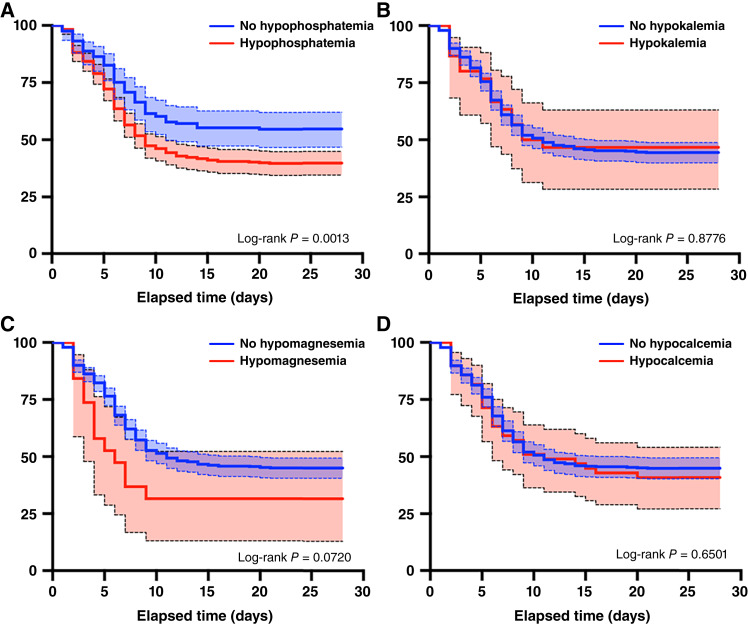
Incidence of ICANS in patients stratified by the presence or absence of electrolyte derangements. Kaplan–Meier analyses comparing time to ICANS for patients stratified by the presence or absence of electrolyte derangements including (**A**) hypophosphatemia, (**B**) hypokalemia, (**C**) hypomagnesemia, and (**D**) hypocalcemia. Shaded area represents the upper and lower bounds of the 95% confidence interval (CI).

We subsequently stratified patients based on the presence or absence of electrolyte derangements following CAR T-cell infusion, including hypophosphatemia, hypokalemia, hypomagnesemia, and hypocalcemia, and performed a time-to-event analysis with respect to ICANS. We found that patients who experienced hypophosphatemia had a significantly higher cumulative incidence of ICANS than those who did not (*P* = 0.0013, log-rank; Fig. 1A). In contrast, the cumulative incidence of ICANS was not significantly different between patients with hypokalemia, hypomagnesemia, or hypocalcemia, compared with patients without the corresponding electrolyte derangements (Fig. 1B–D). Histogram plots of nadir phosphorus values stratified by ICANS status are summarized in Supplementary Fig. S6A–S6D.

### Phosphorus repletion in patients with hypophosphatemia is associated with reduced ICANS incidence and symptom duration

**Figure 2:**
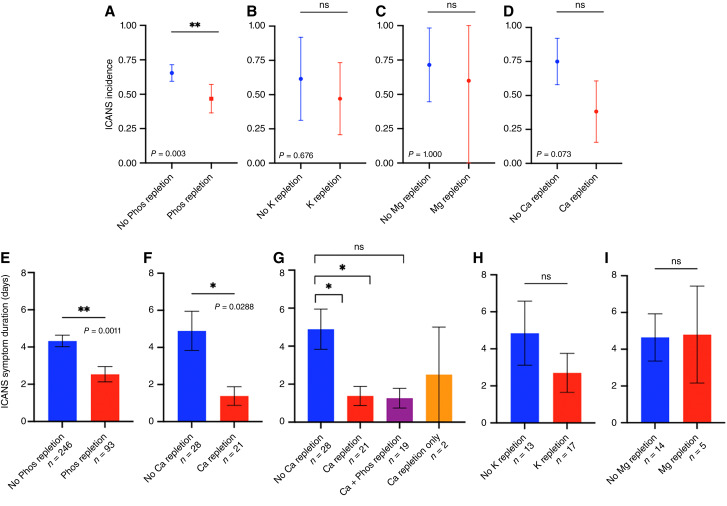
ICANS incidence and symptom duration in patients with various electrolyte derangements stratified by receipt of any repletion of the deficient electrolyte. ICANS incidence (top row, error bars represent 95% confidence interval (CI) in patients with (**A**) serum hypophosphatemia, (**B**) serum hypokalemia, (**C**) serum hypomagnesemia, and (**D**) serum hypocalcemia, stratified by receipt of any electrolyte products containing the deficient electrolyte, from time of CAR T-cell infusion (day 0) until either ICANS symptom onset or day +14. ICANS symptom duration (represented as mean ± SEM) in patients with (**E**) hypophosphatemia or (**F**) hypocalcemia stratified by receipt of any electrolyte products containing the deficient electrolyte, from time of CAR T-cell infusion (day 0) until either ICANS symptom onset or day +14. **G,** ICANS symptom duration in patients with serum hypocalcemia stratified by no repletion (*n* = 28), any calcium repletion (*n* = 21), calcium and phosphorus repletion (*n* = 19), and calcium repletion containing no phosphorus (*n* = 2). **H,** ICANS symptom duration in patients with hypokalemia (**H**) or hypomagnesemia (**I**) stratified by receipt of any electrolyte products containing the deficient electrolyte, from time of CAR T-cell infusion (day 0) until either ICANS symptom onset or day +14. *, *P* < 0.05; **, *P* < 0.01; Fisher exact test for ICANS incidence, unpaired *t* test for ICANS symptom duration.

Given the various electrolyte derangements that occur in the setting of CAR T-cell therapy and the significant association between serum hypophosphatemia and ICANS incidence in our patient cohort, we explored the impact of goal-directed electrolyte supplementation on ICANS incidence and symptom duration. We found that patients with hypophosphatemia who received phosphorus repletion had a significantly lower incidence of ICANS than patients with hypophosphatemia who did not receive phosphorus repletion (46.7% vs. 65.4%, *P* = 0.0030; Fig. 2A). In contrast, goal-directed repletion of potassium, magnesium, and calcium in patients with those corresponding electrolyte deficiencies were not significantly associated with decreased ICANS incidence (Fig. 2B–D). Phosphorus repletion in patients with hypophosphatemia was also associated with a significant decrease in ICANS symptom duration compared with patients with hypophosphatemia who did not receive repletion (2.53 vs. 4.33 days, *P* = 0.0011; Fig. 2E). Interestingly, calcium repletion in patients with hypocalcemia was also significantly associated with decreased ICANS symptom duration (1.38 vs. 4.89 days, *P* = 0.0288; Fig. 2F). However, almost all of the patients with hypocalcemia had concomitant hypophosphatemia, and the majority of these patients received supplementation with both phosphorus and calcium (Supplementary Fig. S7). Indeed, ICANS symptom duration was significantly shorter only in patients with hypocalcemia who received both phosphorus and calcium (*n* = 19, 1.26 days, *P* = 0.0256) and not in patients who received calcium alone (*n* = 2, 2.5 days, *P* = 0.6966), compared with those who did not receive goal-directed repletion (*n* = 28, 4.85 days; Fig. 2G). Goal-directed electrolyte repletion in patients with hypokalemia or hypomagnesemia was not associated with decreased ICANS symptom duration (Fig. 2H and I).

### Electrolyte derangements are associated with distinct subsets of NAEs in CAR T-cell recipients

**Figure 3:**
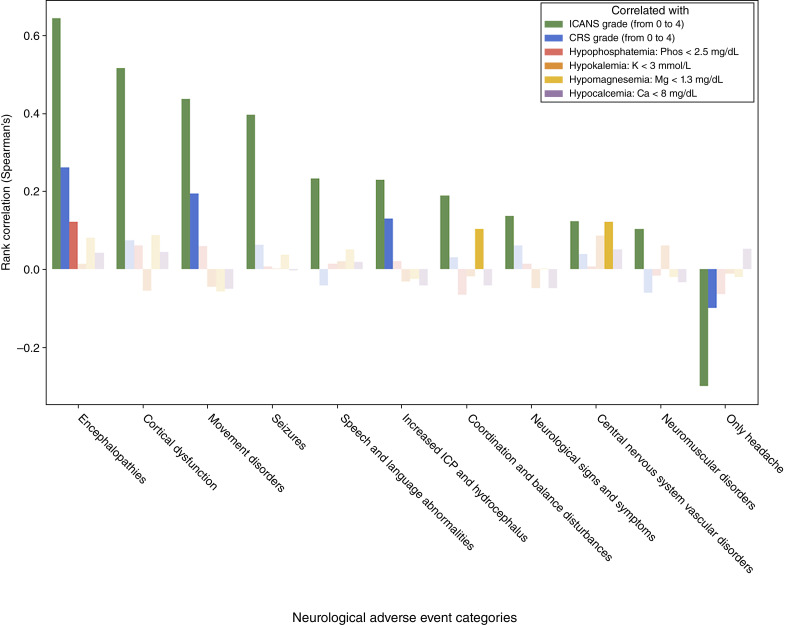
Correlation between ICANS, CRS, and various electrolyte derangements with categories of neurological adverse events reported in CAR T-cell recipients. Data are represented as a bar graph showing Spearman rank correlation. Solid bars represent *P* < 0.05, and translucent bars represent *P* > 0.05.

We further explored the correlation between reported categories of NAEs in CAR T-cell recipients with ICANS and CRS severity, as well as with the various electrolyte derangements. ICANS grade was significantly positively correlated with all other reported categories of NAEs, with the exception of headache alone (Fig. 3). CRS grade was only significantly positively correlated with a subset of NAEs, including encephalopathies, movement disorders, and increased intracranial pressure. The majority of events reported as movement disorders were isolated tremor, and all reports of increased intracranial pressure were secondary to cerebral edema (Supplementary Table S5). Certain electrolyte derangements were also associated with specific subsets of NAEs. Notably, hypophosphatemia was significantly associated with encephalopathies (Spearman’s *r* = 0.1217, *P* = 0.0065), which was also the NAE category that most strongly correlated with ICANS (Spearman’s *r* = 0.6429, *P* = 0.0001; Fig. 3). Included within the higher level “encephalopathies” term are encephalopathy (defined as a pathologic intracranial process that interferes with one’s activities of daily living), somnolence, lethargy, depressed/altered consciousness, memory impairment, mental impairment, cognitive disorder, and disturbances in attention (Supplementary Table S5). Hypomagnesemia was associated with coordination and balance disturbances (Spearman’s *r* = 0.1024, *P* = 0.0222) and central nervous system vascular disorders (Spearman’s *r* = 0.1215, *P* = 0.0065), whereas hypokalemia and hypocalcemia were not significantly associated with any particular NAEs (Fig. 3).

### Anti-CD19 CAR T-cell recipients who develop ICANS have a greater incidence of AKI

Overall, our patient cohort had normal baseline kidney function, as defined by Scr and eGFR; neither of which was significantly different between the ICANS and non-ICANS groups (Supplementary Fig. S8A and S8B). Throughout the course of treatment, there were no significant differences in the change in Scr from baseline between the ICANS and non-ICANS groups on post-infusion days 5 to 7 and days 14 to 15 (Supplementary Fig. S8C). There were also no significant differences in eGFR changes from baseline between the two groups on post-infusion days 5 to 7; however, the ICANS group was found to have a higher *increase* in their eGFR from baseline on post-infusion days 14 to 15 (Supplementary Fig. S8D). The incidence of any stage AKI was 16.8% in the overall cohort, including 11.4% with stage 1, 2.2% with stage 2, and 3.2% with stage 3 (Table 1). In the ICANS group, the incidence of AKI was two-fold higher than in the non-ICANS group (21.7% vs. 10.8% *P* = 0.0016; Supplementary Fig. S8E). These differences in AKI incidence may be reflected in the relatively higher number of outliers in the ICANS group than the non-ICANS group, who had higher increases in their creatinine and decreases in their eGFR compared with their baseline (Supplementary Fig. S8C and S8D). We observed no significant difference in the incidence of hypophosphatemia in patients who developed AKI compared with those who did not (Supplementary Table 6). In the patients who developed both AKI and hypophosphatemia, we observed that the AKI occurred after the onset of hypophosphatemia, with a median onset of AKI 3 days after the onset of hypophosphatemia (95% CI, 1–4 days).

## Discussion

ICANS occurs in more than half of CAR T-cell recipients and poses significant clinical challenges given its morbidity and limited treatment options (3). Anakinra, an IL1 antagonist, has been investigated in clinical trials for the treatment and prophylaxis of ICANS based on preclinical models implicating IL1 production by host monocytes as a possible driver (14). Although studies are still ongoing, anakinra treatment has thus far not demonstrated any significant reductions in neurologic symptom severity or duration in refractory ICANS (15, 16). Furthermore, in early phase trials exploring anakinra for ICANS prophylaxis, a significant proportion of patients continued to develop ICANS (17). These clinical outcomes highlight an ongoing need for effective interventions for ICANS.

In this study, we showed that serum hypophosphatemia was a common occurrence in the setting of CAR T-cell therapy and was associated with increased cumulative incidence of ICANS. Moreover, phosphorus repletion in patients with hypophosphatemia was associated with decreased ICANS incidence and symptom duration. We also found that hypophosphatemia was uniquely associated with encephalopathic symptoms, which was also the NAE category that most strongly correlated with ICANS.

Hypomagnesemia was the only other electrolyte derangement found to correlate with any NAE categories. Although magnesium is thought to be neuroprotective and its deficiency has been linked to ataxia symptoms, given the low incidence of hypomagnesemia in our cohort, it is difficult to determine whether these associations are a direct result of magnesium deficiency or if the presence of hypomagnesemia reflects a more critically ill subpopulation vulnerable to these NAEs (18). Interestingly, CRS severity was associated with encephalopathy and movement disorders, the latter category representing almost exclusively isolated symptoms of tremor in our cohort; however, it was also uniquely associated with cerebral edema—a feature of severe ICANS with a fulminant onset and few antecedent warning signs that seems to deviate from the classic progression of symptoms (3). It is possible that distinct pathophysiologic mechanisms contribute to different NAEs, and hypophosphatemia in the setting of CAR T-cell therapy may contribute to the encephalopathic manifestations. Additionally, the similarity in symptoms between hypophosphatemia and ICANS may potentially lead to diagnostic confusion, especially in low-grade ICANS. Thus, at minimum, the expeditious goal-directed correction of electrolyte derangements may unmask any clinical confounders and identify patients at risk for more serious neurological sequelae.

Multiple groups, including ours, have reported electrolyte derangements like hypophosphatemia occurring in the setting of CAR T-cell therapy, yet the precise mechanisms remain unclear. Given the presence of strong homeostatic mechanisms to maintain blood phosphorus within the normal range, small deviations of phosphorus levels from the normal set point represent a failure of the compensatory mechanisms that regulate its level, which may include shifts in intracellular and extracellular phosphorus levels or between tissues (e.g., mineral bone). Although the absolute differences between nadir phosphorus values for patients who do or do not experience ICANS were small, these differences may represent a more extreme and abnormal physiological state in those individuals who experience ICANS, which is manifested in part by the emergence of neurological symptoms. Although the baseline renal function in our cohort was within normal limits, AKI has been shown to occur in a significant proportion of CAR T-cell recipients (19). This is likely a consequence of CRS, as hemodynamic changes driven by massive systemic inflammation lead to decreased renal perfusion (19). The overall incidence of AKI in our cohort was 16.8% and was two-fold higher in those who developed ICANS. Interestingly, AKI with decreased GFR usually results in hyperphosphatemia, yet phosphate concentrations were significantly lower in the ICANS group. Furthermore, the observation that hypophosphatemia incidence was no different in patients who developed AKI, and occurred typically prior to the onset of AKI, further strengthens the argument that the development of AKI does not contribute to hypophosphatemia in the setting of CAR T-cell infusion. Indeed, AKI would actually decrease renal excretion of phosphorus, suggesting that hypophosphatemia is happening in spite of AKI, rather than because of it.

Renal phosphate wasting independent of GFR changes can occur with proximal tubulopathies caused by direct cytokine-induced proximal tubule injury. Alternatively, in the setting of systemic inflammation, an IL6-driven upregulation of fibroblast growth factor 23 could lead to increased renal phosphate excretion and changes in hormones involved in phosphate homeostasis (20). In one study, however, a decrease in fibroblast growth factor 23 was observed following CAR T-cell infusion, with a corresponding increase in activated vitamin D3 and unchanged parathyroid hormone levels (8). Moreover, multiple groups have reported stable serum calcium levels in the setting of pronounced hypophosphatemia (8, 19). Taken together, the acute hypophosphatemia following CAR T-cell infusion is unlikely a result of renally dysregulated phosphorus homeostasis. In fact, the data from our cohort show an appropriate homeostatic response attempting to compensate for this state of hypophosphatemia. In future studies, additional biomarkers including urinary proximal tubule–derived proteins and urinary electrolytes pre- and post-CAR T-cell infusion can help clarify the extent mechanism of any renal involvement.

Hypophosphatemia has also been well described in significantly metabolically active states such as sepsis as a result of intracellular redistribution of phosphorus driven by the increased need for phosphorus-dependent metabolic intermediates (9, 10). CAR T-cell infusion is a potent driver of systemic inflammation, evidenced by significant elevations in inflammatory markers and serum cytokine levels, often manifesting as CRS (1). Additionally, the demand for phosphorylated intermediates by the metabolically active infusion product may drive an intracellular redistribution of phosphorus that results in hypophosphatemia. Indeed, our group has previously demonstrated *in vitro* that CAR T-cell effector activity is associated with an uptake of extracellular phosphate, which is temporally associated with increased phosphorus-dependent metabolism driven by target antigen engagement (7). In the current study, serum phosphorus concentrations declined rapidly following CAR T-cell infusion and reached nadir values in most patients between day 5 and day 7, coinciding with both the reported peak expansion of the infusion product and the onset of ICANS symptoms in our cohort. Conditioned on the presence of CRS, we observed no significant difference in the rates of hypophosphatemia for patients who do or do not experience ICANS. This finding supports the interpretation that CRS significantly increases the risk of ICANS by a variety of mechanisms, including, but not limited to, electrolyte disturbances.

To our knowledge, this is the first study to report an association between electrolyte derangements and specific categories of neurological manifestations in CAR T-cell recipients and examine the effects of goal-directed electrolyte repletion on ICANS incidence and symptom duration. The retrospective nature of this study limits our ability to ascribe a causative relationship between hypophosphatemia and ICANS. Additionally, the lack of standardized protocols for electrolyte repletion and variation with respect to the dose and duration of any repletion given further limits our interpretation and ability to extrapolate ideal thresholds and regimens for interventional electrolyte repletions. Indeed, the potentially multifactorial nature of ICANS pathogenesis would require highly multidimensional datasets unavailable in our study, such as myeloid cell–associated serum cytokines, as well as markers of endothelial activation and/or blood–brain barrier disruption, which could both contribute to the hypophosphatemia or the development of ICANS itself (21). Finally, our study was limited by the lack of any pediatric patients or 4-1BB co-stimulatory domain CAR T-cell products, as well as lack of consistent information regarding cell dosage received by the patients.

In summary, these results suggest that serum phosphorus could be a reliable biomarker to trend in CAR T-cell recipients while monitoring for ICANS. Our data also demonstrate that phosphorus repletion in response to hypophosphatemia could be a safe, inexpensive, and widely available intervention for some of the frequently encountered symptoms of ICANS. Future studies aimed at elucidating the causative mechanisms between hypophosphatemia and ICANS, as well as prospective clinical protocols to evaluate phosphorus repletion for the prophylaxis and management of ICANS will be important in advancing this growing area of specialized care of patients receiving cellular immunotherapies.

## Supplementary Material

Supplemental Table 1 Neurological adverse events included within each neurological adverse event category for CAR T-cell recipients.

Supplemental Table 2 Range and interquartile range of nadir serum electrolyte values in patients with and without ICANS.

Supplemental Table 3 Incidence of hypophosphatemia and ICANS in patients who developed CRS (n = 433).

Supplemental Table 4 Stratification of CRS, ICANS, and hypophosphatemia incidence rates by pre-emptive treatment with tocilizumab.

Supplemental Table 5 Correlation between ICANS, CRS, and various electrolyte derangements with categories of neurological adverse events reported in CAR T-cell recipients.

Supplemental Table 6 Incidence of hypophosphatemia and AKI following CAR-T cell infusion.

Figure S1 Figure S1. Nadir serum electrolyte concentrations grouped by patient ICANS status.

Figure S2 Figure S2. Trends in serum electrolyte concentrations following CAR T-cell infusion grouped by patient ICANS status.

Figure S3 Figure S3. ICANS incidence and time to symptom onset.

Figure S4 Figure S4. Association of hypophosphatemia incidence by ICANS grade.

Figure S5 Figure S5. Associations between pre-infusion tumor burden and ICANS and hypophosphatemia.

Figure S6 Figure S6. Histogram plots of nadir phosphorus values stratified by ICANS status.

Figure S7 Figure S7. Coincidence of serum hypocalcemia with hypophosphatemia.

Figure S8 Figure S8. Baseline and change in kidney function following CAR T therapy grouped by patient ICANS status.

Supplemental Figure Legends Supplemental Figure Legends
