# Supplementary material for: Hypophosphatemia Correction Reduces ICANS Incidence and Duration in CAR T-cell Therapy: A Pooled Clinical Trial Analysis
Source: Cancer Res Commun. 2024 Oct 3;4(10):2589–97. doi: 10.1158/2767-9764.CRC-24-0250 (PMC11448391; doi:10.1158/2767-9764.CRC-24-0250)
Supplement: Supplemental Table 1 — Neurological adverse events included within each neurological adverse event category for CAR T-cell recipients. [file crc-24-0250_supplemental_table_1_suppst1.docx]

**Supplemental Table 1. Neurological adverse events included within each neurological adverse event category for CAR T-cell recipients**

| **MedDRA High Level Group Term (Neurological Adverse Event Categories)** | **MedDRA Preferred Term (Neurological Adverse Event)** | **Number of Patients** |
| --- | --- | --- |
| Encephalopathies | CAR T-cell-related encephalopathy syndrome | 6 |
|  | Metabolic encephalopathy | 1 |
|  | Toxic encephalopathy | 1 |
|  | Encephalopathy | 154 |
|  | Leukoencephalopathy | 2 |
|  | Somnolence | 87 |
|  | Lethargy | 34 |
|  | Syncope | 15 |
|  | Depressed level of consciousness | 14 |
|  | Stupor | 2 |
|  | Altered state of consciousness | 1 |
|  | Loss of consciousness | 1 |
|  | Memory impairment | 31 |
|  | Cognitive disorder | 17 |
|  | Disturbance in attention | 13 |
|  | Amnesia | 8 |
|  | Mental impairment | 3 |
| Cortical Dysfunction | Aphasia | 114 |
|  | Dysgraphia | 10 |
|  | Apraxia | 5 |
|  | Dyscalculia | 1 |
|  | Dyspraxia | 1 |
| Movement Disorders | Tremor | 171 |
|  | Head titubation | 1 |
|  | Hemiparesis | 4 |
|  | Paresis | 1 |
|  | Quadriplegia | 1 |
|  | Dyskinesia | 7 |
|  | Psychomotor hyperactivity | 3 |
|  | Motor dysfunction | 2 |
|  | Fine motor skill dysfunction | 1 |
|  | Hypokinesia | 1 |
| Seizures | Seizure | 31 |
|  | Partial seizures | 2 |
|  | Seizure like phenomena | 1 |
|  | Status epilepticus | 1 |
|  | Generalized tonic-clonic seizure | 1 |
|  | Petit mal epilepsy | 1 |
| Speech and Language Abnormalities | Dysarthria | 25 |
|  | Speech disorder | 4 |
|  | Slow speech | 1 |
| Increased ICP | Brain oedema | 8 |
| Coordination and Balance Disturbances | Ataxia | 16 |
|  | Nystagmus | 6 |
|  | Dysmetria | 4 |
|  | Balance disorder | 2 |
|  | Coordination abnormal | 1 |
|  | Dysdiadochokinesis | 1 |
| Neurological Signs and Symptoms | Dizziness | 105 |
|  | Presyncope | 8 |
|  | Myoclonus | 6 |
|  | Slow response to stimuli | 2 |
|  | Dizziness postural | 1 |
|  | Head discomfort | 1 |
|  | Meningism | 1 |
|  | Neurological decompensation | 1 |
|  | Neurological symptom | 1 |
| CNS Vascular Disorders | Transient ischemic attack | 1 |
|  | Cerebral venous sinus thrombosis | 1 |
|  | Cerebrovascular accident | 4 |
|  | Cerebellar infarction | 2 |
|  | Hemorrhage intracranial | 2 |
|  | Cerebral infarction | 1 |
|  | Cerebral ischemia | 1 |
|  | Embolic stroke | 1 |
| Neuromuscular Disorders | Muscle contractions involuntary | 3 |
|  | Muscle spasticity | 1 |
|  | Neuromyopathy | 1 |
| Headache | Headache | 277 |

Symptoms in red were originally classified under disturbances in consciousness and symptoms in blue were originally classified under mental impairment disorders. These have been included within the higher-level encephalopathies category given the clinical overlap between the lower-level terms.
