# Supplementary material for: Hypophosphatemia Correction Reduces ICANS Incidence and Duration in CAR T-cell Therapy: A Pooled Clinical Trial Analysis
Source: Cancer Res Commun. 2024 Oct 3;4(10):2589–97. doi: 10.1158/2767-9764.CRC-24-0250 (PMC11448391; doi:10.1158/2767-9764.CRC-24-0250)
Supplement: Supplemental Table 2 — Range and interquartile range of nadir serum electrolyte values in patients with and without ICANS. [file crc-24-0250_supplemental_table_2_suppst2.docx]

**Supplemental Table 2 . Range and interquartile range of nadir serum electrolyte values in patients with and without ICANS.**

| Nadir Phos (mg/dL) | N | Mean | SD | Min | 25%ile | 50%ile | 75%ile | Max | IQR |
| --- | --- | --- | --- | --- | --- | --- | --- | --- | --- |
| NO ICANS | 222 | 2.2 | 0.72910 | 0.558 | 1.7 | 2.2 | 2.7 | 4.20546 | 1.00 |
| ICANS | 277 | 2.0 | 0.62192 | 0.50065 | 1.7 | 2.0 | 2.5 | 3.90507 | 0.81 |
|  |  |  |  |  |  |  |  |  |  |
| Nadir CA (mg/dL) | **N** | **Mean** | **SD** | **Min** | **25%ile** | **50%ile** | **75%ile** | **Max** | **IQR** |
| NO ICANS | 222 | 8.59 | 0.48470 | 7.06 | 8.3 | 8.582 | 8.9 | 10.224 | 0.6 |
| ICANS | 277 | 8.58 | 0.50811 | 7.08 | 8.28 | 8.56 | 8.84 | 10.6 | 0.56 |
|  |  |  |  |  |  |  |  |  |  |
| Nadir K (mmol/L) | **N** | **Mean** | **SD** | **Min** | **25%ile** | **50%ile** | **75%ile** | **Max** | **IQR** |
| NO ICANS | 222 | 3.46 | 0.31670 | 2.4 | 3.3 | 3.5 | 3.7 | 4.2 | 0.4 |
| ICANS | 277 | 3.41 | 0.30222 | 2.5 | 3.2 | 3.4 | 3.6 | 4.5 | 0.4 |
|  |  |  |  |  |  |  |  |  |  |
| Nadir MG (mg/dL) | **N** | **Mean** | **SD** | **Min** | **25%ile** | **50%ile** | **75%ile** | **Max** | **IQR** |
| NO ICANS | 222 | 1.70 | 0.17799 | 1.1 | 1.6 | 1.70 | 1.8 | 2.09937 | 0.2 |
| ICANS | 277 | 1.65 | 0.18636 | 0.95 | 1.5 | 1.67 | 1.8 | 2.19934 | 0.3 |
