# Supplementary material for: Hypophosphatemia Correction Reduces ICANS Incidence and Duration in CAR T-cell Therapy: A Pooled Clinical Trial Analysis
Source: Cancer Res Commun. 2024 Oct 3;4(10):2589–97. doi: 10.1158/2767-9764.CRC-24-0250 (PMC11448391; doi:10.1158/2767-9764.CRC-24-0250)
Supplement: Supplemental Table 3 — Incidence of hypophosphatemia and ICANS in patients who developed CRS (n = 433). [file crc-24-0250_supplemental_table_3_suppst3.docx]

**Supplemental Table 3. Incidence of hypophosphatemia and ICANS in patients who developed CRS (n=433)**

|  | **Hypophosphatemia -** | **Hypophosphatemia +** |
| --- | --- | --- |
| **ICANS -** | 56 | 122 |
| **ICANS +** | 67 | 198 |

p = 0.16 (Fisher’s exact test)
