# Supplementary material for: Hypophosphatemia Correction Reduces ICANS Incidence and Duration in CAR T-cell Therapy: A Pooled Clinical Trial Analysis
Source: Cancer Res Commun. 2024 Oct 3;4(10):2589–97. doi: 10.1158/2767-9764.CRC-24-0250 (PMC11448391; doi:10.1158/2767-9764.CRC-24-0250)
Supplement: Supplemental Table 4 — Stratification of CRS, ICANS, and hypophosphatemia incidence rates by pre-emptive treatment with tocilizumab. [file crc-24-0250_supplemental_table_4_suppst4.docx]

**Supplemental Table 4. Stratification of CRS, ICANS, and hypophosphatemia incidence rates by pre-emptive treatment with tocilizumab.**

|  | CRS - | CRS + | **Rate of CRS** |
| --- | --- | --- | --- |
| **Pre-emptive tocilizumab +** | 18 | 116 | 86.6% |
| **Pre-emptive tocilizumab -** | 28 | 337 | 92.3% |

p = 0.055 (Fisher’s exact test)

|  | Hypophosphatemia - | Hypophosphatemia + | **Rate of hypophosphatemia** |
| --- | --- | --- | --- |
| **Pre-emptive tocilizumab +** | 57 | 77 | 57.5% |
| **Pre-emptive tocilizumab -** | 104 | 261 | 71.5% |

p = 0.0035 (Fisher’s exact test)

|  | ICANS - | ICANS + | **Rate of ICANS** |
| --- | --- | --- | --- |
| **Pre-emptive tocilizumab +** | 56 | 78 | 58.2% |
| **Pre-emptive tocilizumab -** | 166 | 199 | 54.5% |

p = 0.48 (Fisher’s exact test)

Pre-emptive tocilizumab group (n = 134)

|  | CRS - | CRS + | **Rate of CRS** |
| --- | --- | --- | --- |
| **Hypophosphatemia -** | 12 | 45 | 78.9% |
| **Hypophosphatemia +** | 6 | 71 | 92.2% |

p = 0.0388 (Fisher’s exact test)

Pre-emptive tocilizumab group (n = 134)

|  | **No ICANS** | **ICANS** | **Rate of ICANS** |
| --- | --- | --- | --- |
| **Hypophosphatemia -** | 30 | 27 | 47.4% |
| **Hypophosphatemia +** | 26 | 51 | 66.2% |

p = 0.0343 (Fisher’s exact test)

No Pre-emptive tocilizumab group (n = 365)

|  | **No CRS** | **CRS** | **Rate of CRS** |
| --- | --- | --- | --- |
| **Hypophosphatemia -** | 19 | 85 | 81.7% |
| **Hypophosphatemia +** | 9 | 252 | 96.6% |

p < 0.0001 (Fisher’s exact test)

No Pre-emptive tocilizumab group (n = 365)

|  | **No ICANS** | **ICANS** | **Rate of ICANS** |
| --- | --- | --- | --- |
| **Hypophosphatemia -** | 58 | 46 | 44.2% |
| **Hypophosphatemia +** | 108 | 153 | 58.6% |

p = 0.0145 (Fisher’s exact test)
