# Supplementary material for: Hypophosphatemia Correction Reduces ICANS Incidence and Duration in CAR T-cell Therapy: A Pooled Clinical Trial Analysis
Source: Cancer Res Commun. 2024 Oct 3;4(10):2589–97. doi: 10.1158/2767-9764.CRC-24-0250 (PMC11448391; doi:10.1158/2767-9764.CRC-24-0250)
Supplement: Supplemental Table 5 — Correlation between ICANS, CRS, and various electrolyte derangements with categories of neurological adverse events reported in CAR T-cell recipients. [file crc-24-0250_supplemental_table_5_suppst5.docx]

**Supplemental Table 5. Correlation between ICANS, CRS, and various electrolyte derangements with categories of neurological adverse events reported in CAR T-cell recipients.**

| **Neurologic Adverse Event** | **ICANS Grade** | **CRS Grade** | **Hypophosphatemia** | **Hypokalemia** | **Hypomagnesemia** | **Hypocalcemia** |
| --- | --- | --- | --- | --- | --- | --- |
| Encephalopathies | **0.6429**  **(<0.0001)** | **0.2615**  **(<0.0001)** | **0.1217**  **(0.0065)** | 0.0128 (0.7751) | 0.0812  (0.0700) | 0.0424 (0.3446) |
| Cortical dysfunction | **0.5149**  **(<0.0001)** | 0.0746  (0.0959) | 0.0606  (0.1765) | -0.056 (0.2120) | 0.0876  (0.0504) | 0.0433 (0.3343) |
| Movement disorders | **0.4363**  **(<0.0001)** | **0.1935**  **(<0.0001)** | 0.0596  (0.1841) | -0.0463 (0.3024) | -0.0577  (0.1980) | -0.0514 (0.2514) |
| Seizures | **0.3963 (<0.0001)** | 0.0617 (0.1688) | 0.0066  (0.8833) | 0.0024 (0.9571) | 0.0377  (0.4012) | -0.0037 (0.9335) |
| Speech and language abnormalities | **0.2319 (<0.0001)** | -0.0424 (0.3447) | 0.014  (0.7556) | 0.02 (0.6550) | 0.0508  (0.2575) | 0.0192 (0.6693) |
| Increased intracranial pressure and hydrocephalus | **0.2289 (<0.0001)** | **0.1299 (0.0037)** | 0.0196  (0.6629) | -0.0323 (0.4718) | -0.0254  (0.5714) | -0.0421 (0.3478) |
| Coordination and balance disturbances | **0.1881 (<0.0001)** | 0.0306 (0.4952) | -0.0654  (0.1445) | -0.0182 (0.6845) | **0.1024**  **(0.0222)** | -0.0419 (0.3507) |
| Neurological signs and symptoms | **0.1368 (0.0022)** | 0.0613 (0.1714) | 0.0137  (0.76) | -0.0493 (0.2719) | 0.0023  (0.9596) | -0.0497 (0.2676) |
| Central nervous system vascular disorders | **0.1229 (0.0060)** | 0.0388 (0.3876) | 0.0072  (0.873) | 0.0851 (0.0575) | **0.1216**  **(0.0065)** | 0.0497 (0.2676) |
| Neuromuscular disorders | **0.1034 (0.0209)** | -0.0608 (0.1752) | -0.0165  (0.7135) | 0.0599 (0.1817) | -0.02  (0.6556) | -0.0332 (0.4593) |
| Only Headache | **-0.3007 (<0.0001)** | **-0.0997 (0.0259)** | -0.0646  (0.1494) | -0.0126 (0.7794) | -0.0202  (0.6531) | 0.0514 (0.2519) |

Values presented as Spearman’s rank correlation with p-value in parentheses.

p-values <0.5 are bolded.
