# Supplementary material for: Hypophosphatemia Correction Reduces ICANS Incidence and Duration in CAR T-cell Therapy: A Pooled Clinical Trial Analysis
Source: Cancer Res Commun. 2024 Oct 3;4(10):2589–97. doi: 10.1158/2767-9764.CRC-24-0250 (PMC11448391; doi:10.1158/2767-9764.CRC-24-0250)
Supplement: Supplemental Table 6 — Incidence of hypophosphatemia and AKI following CAR-T cell infusion. [file crc-24-0250_supplemental_table_6_suppst6.docx]

**Supplemental Table 6. Incidence of hypophosphatemia and AKI following CAR-T cell infusion.**

|  | **AKI +** | **AKI -** |
| --- | --- | --- |
| **Hypophosphatemia -** | 5 | 156 |
| **Hypophosphatemia +** | 17 | 321 |

p = 0.48 (Fisher’s exact test)
