# Supplementary figures and images for: Hypophosphatemia Correction Reduces ICANS Incidence and Duration in CAR T-cell Therapy: A Pooled Clinical Trial Analysis
Source: Cancer Res Commun. 2024 Oct 3;4(10):2589–97. doi: 10.1158/2767-9764.CRC-24-0250 (PMC11448391; doi:10.1158/2767-9764.CRC-24-0250)

## Slide 1
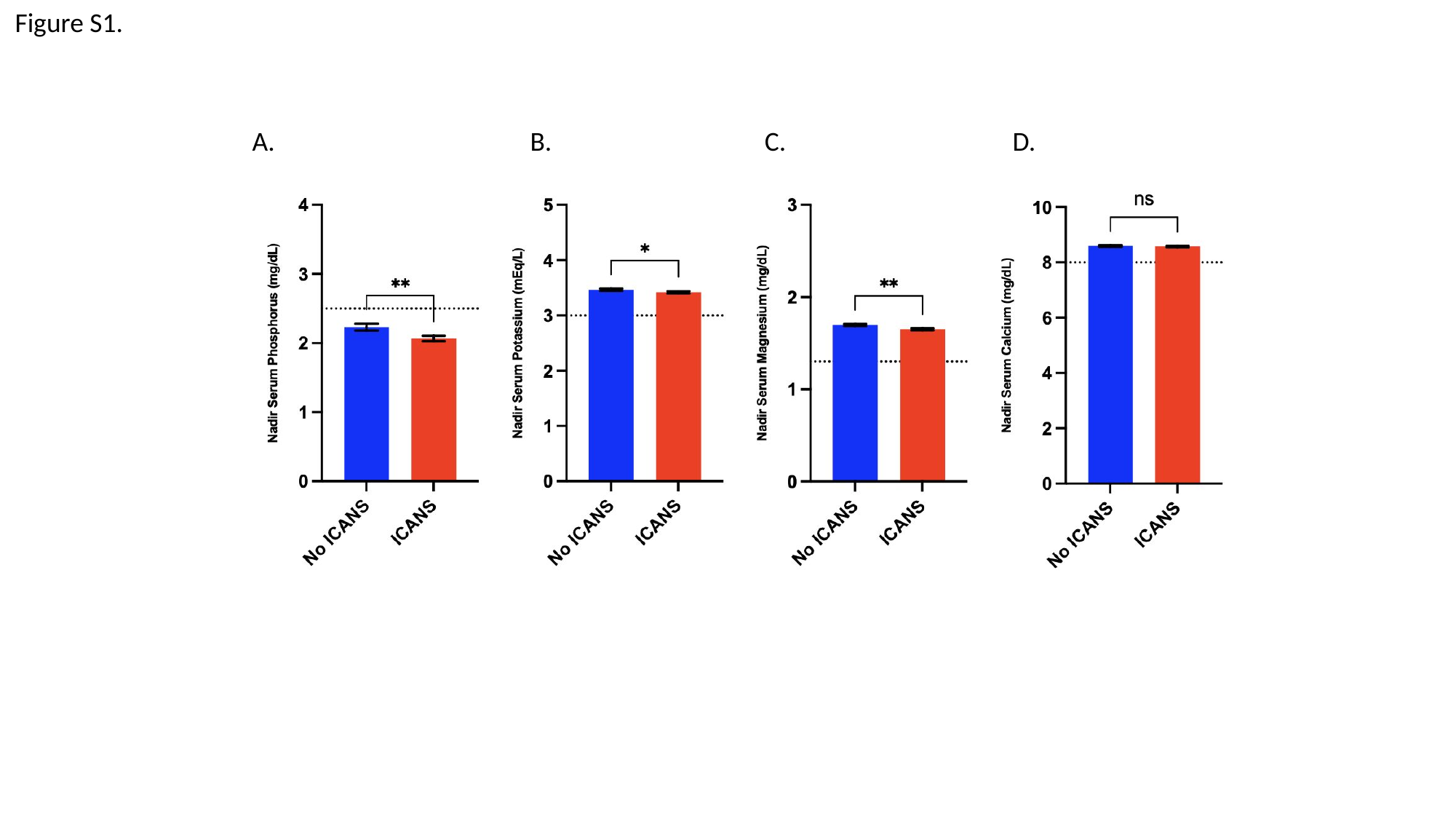

Figure S1.
A.
B.
C.
D.

Supplement: Figure S1 — Nadir serum electrolyte concentrations grouped by patient ICANS status. [file crc-24-0250_figure_s1_suppsf1.pptx]

## Slide 1
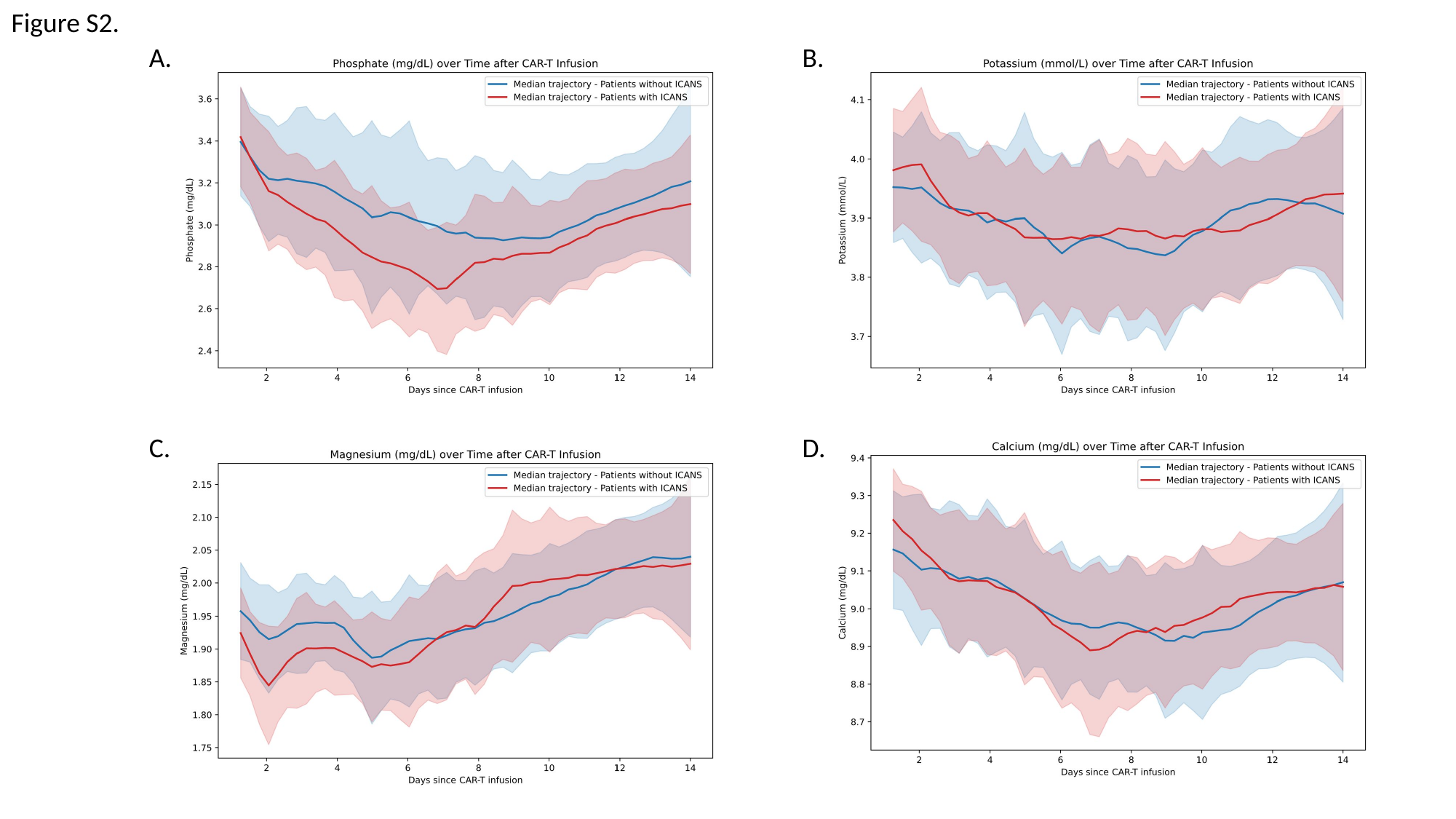

Figure S2.
A.
B.
C.
D.

Supplement: Figure S2 — Trends in serum electrolyte concentrations following CAR T-cell infusion grouped by patient ICANS status. [file crc-24-0250_figure_s2_suppsf2.pptx]

## Slide 1
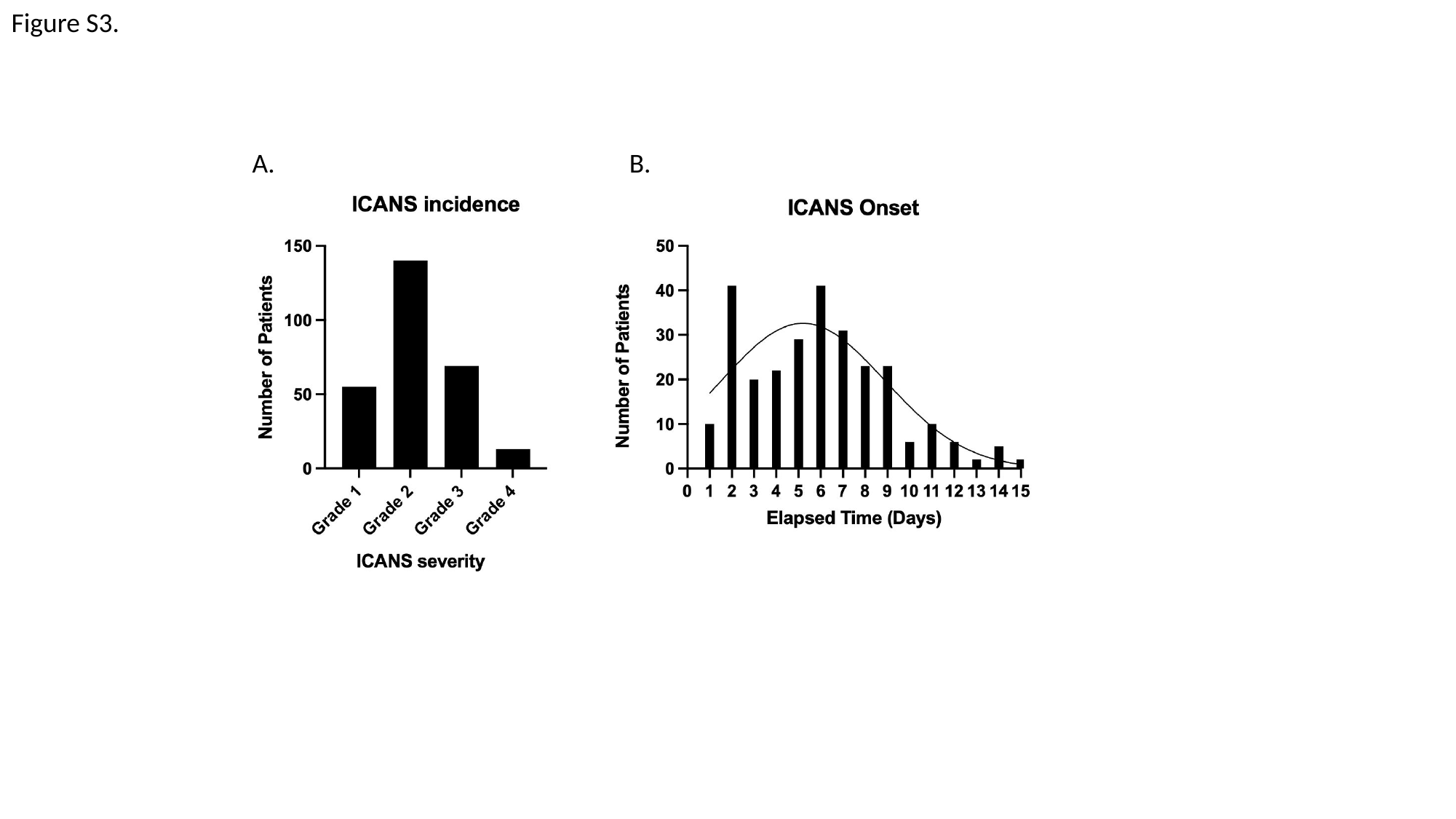

Figure S3.
A.
B.

Supplement: Figure S3 — ICANS incidence and time to symptom onset. [file crc-24-0250_figure_s3_suppsf3.pptx]

## Slide 1
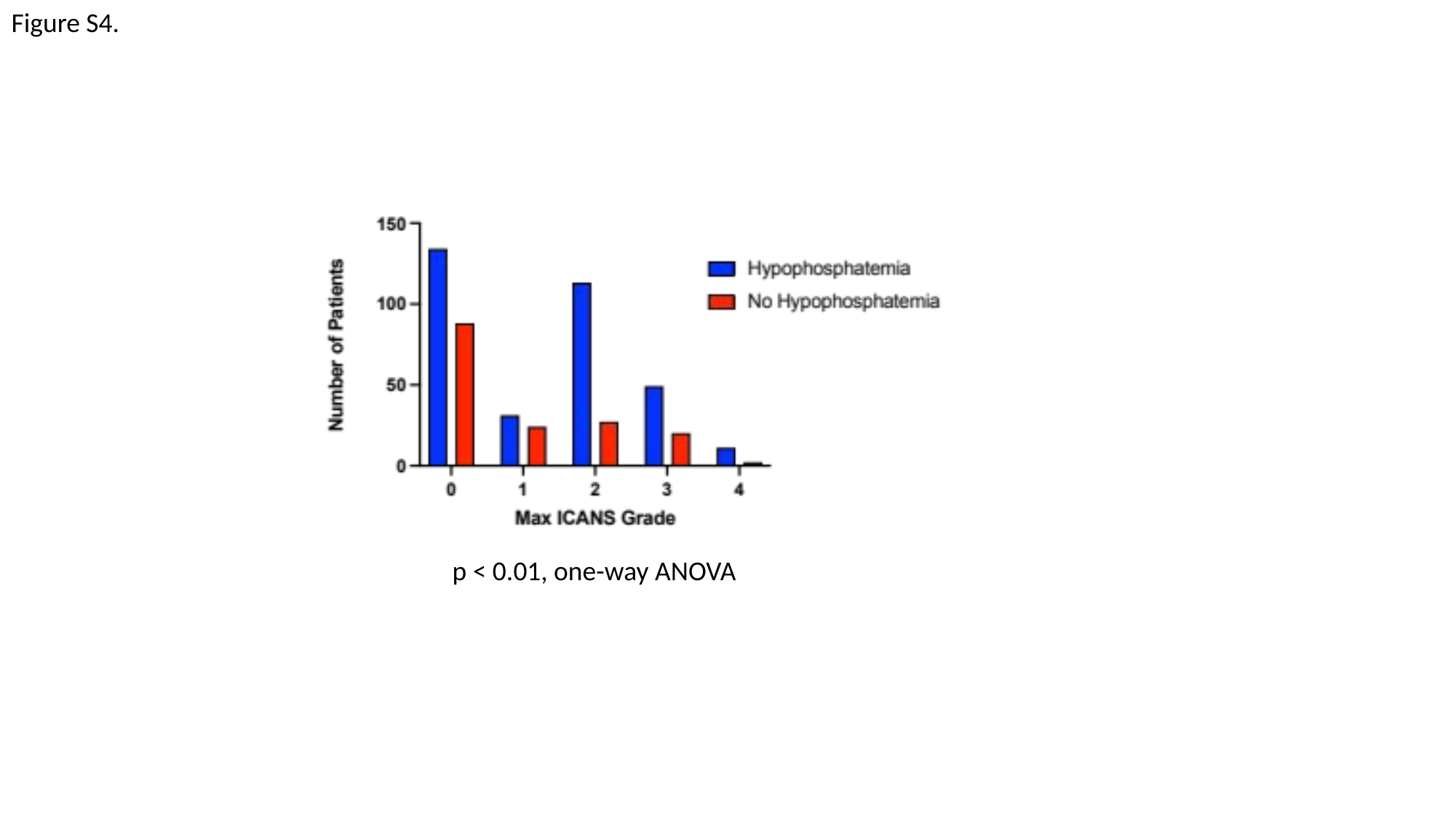

Figure S4.
p < 0.01, one-way ANOVA

Supplement: Figure S4 — Association of hypophosphatemia incidence by ICANS grade. [file crc-24-0250_figure_s4_suppsf4.pptx]

## Slide 1
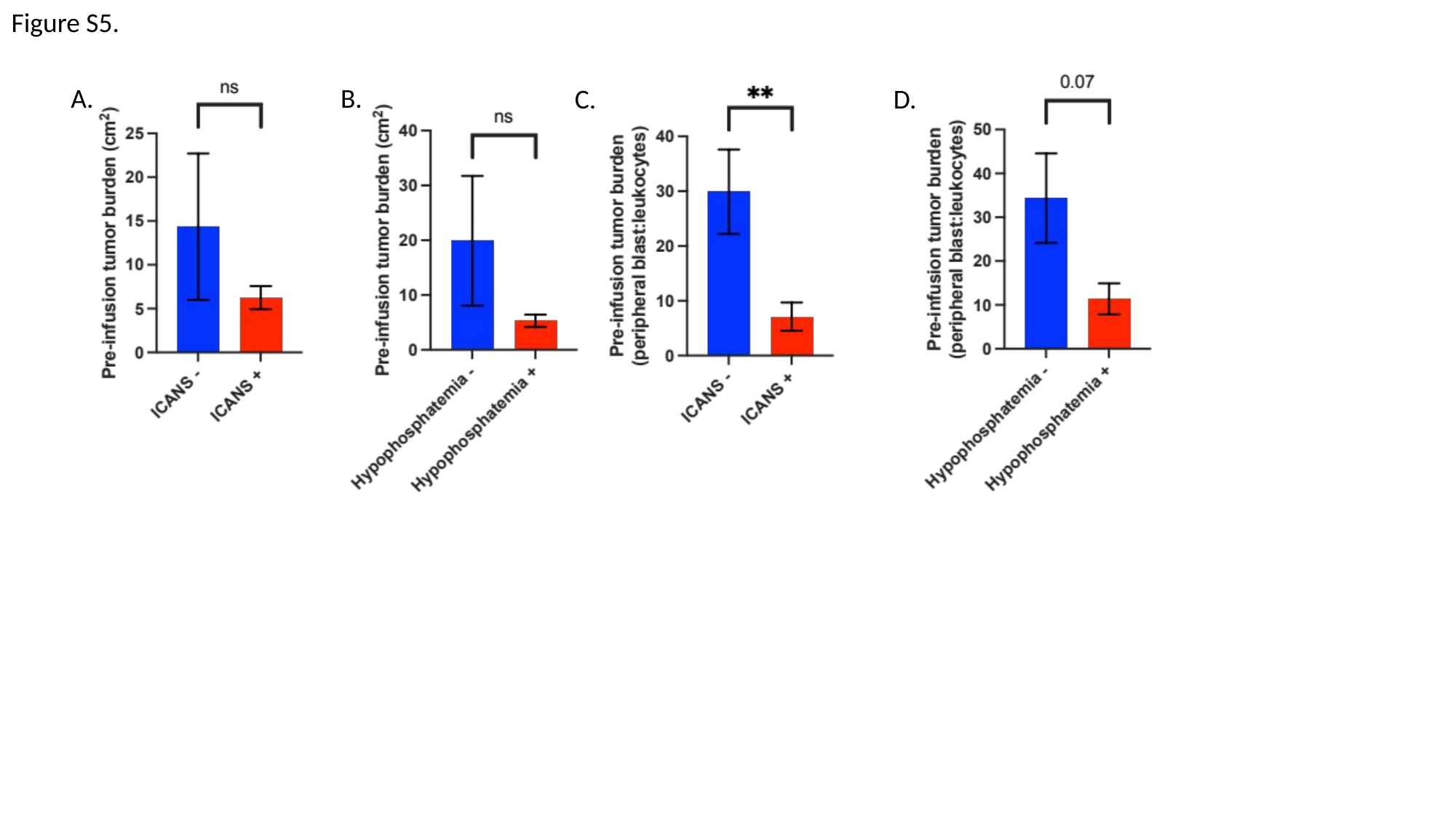

Figure S5.
A.
B.
C.
D.

Supplement: Figure S5 — Associations between pre-infusion tumor burden and ICANS and hypophosphatemia. [file crc-24-0250_figure_s5_suppsf5.pptx]

## Slide 1
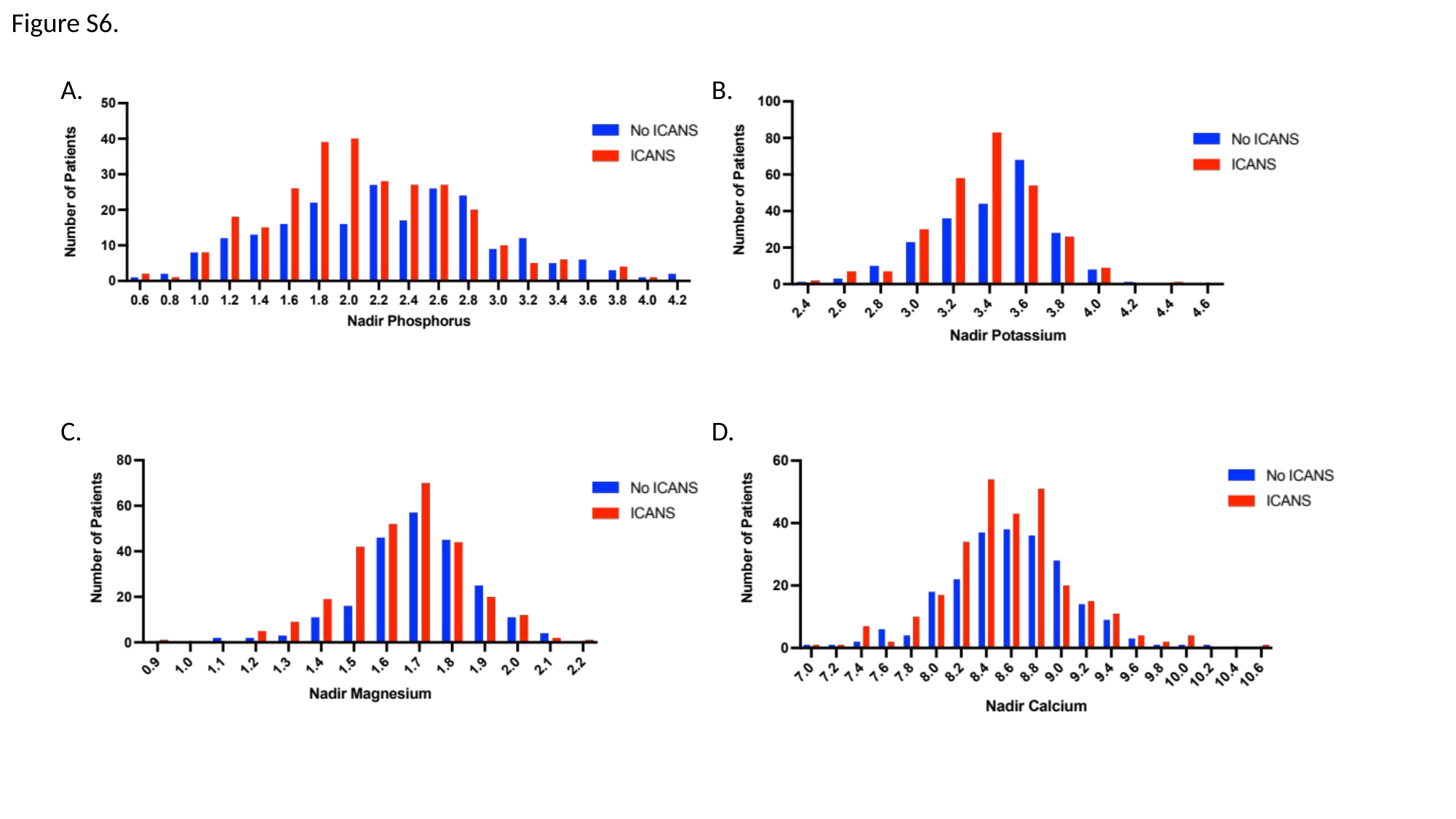

Figure S6.
A.
B.
C.
D.

Supplement: Figure S6 — Histogram plots of nadir phosphorus values stratified by ICANS status. [file crc-24-0250_figure_s6_suppsf6.pptx]

## Slide 1
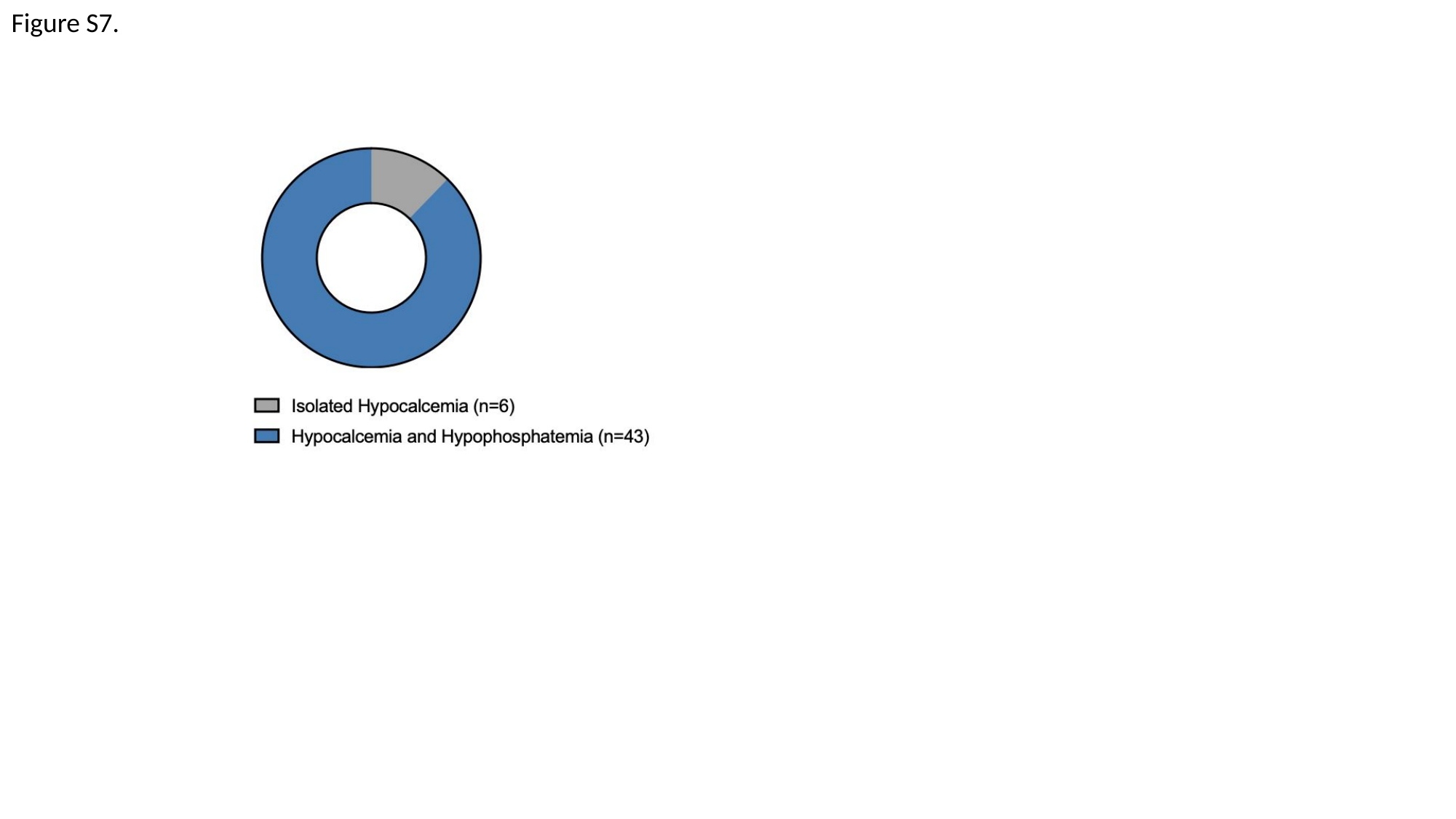

Figure S7.

Supplement: Figure S7 — Coincidence of serum hypocalcemia with hypophosphatemia. [file crc-24-0250_figure_s7_suppsf7.pptx]

## Slide 1
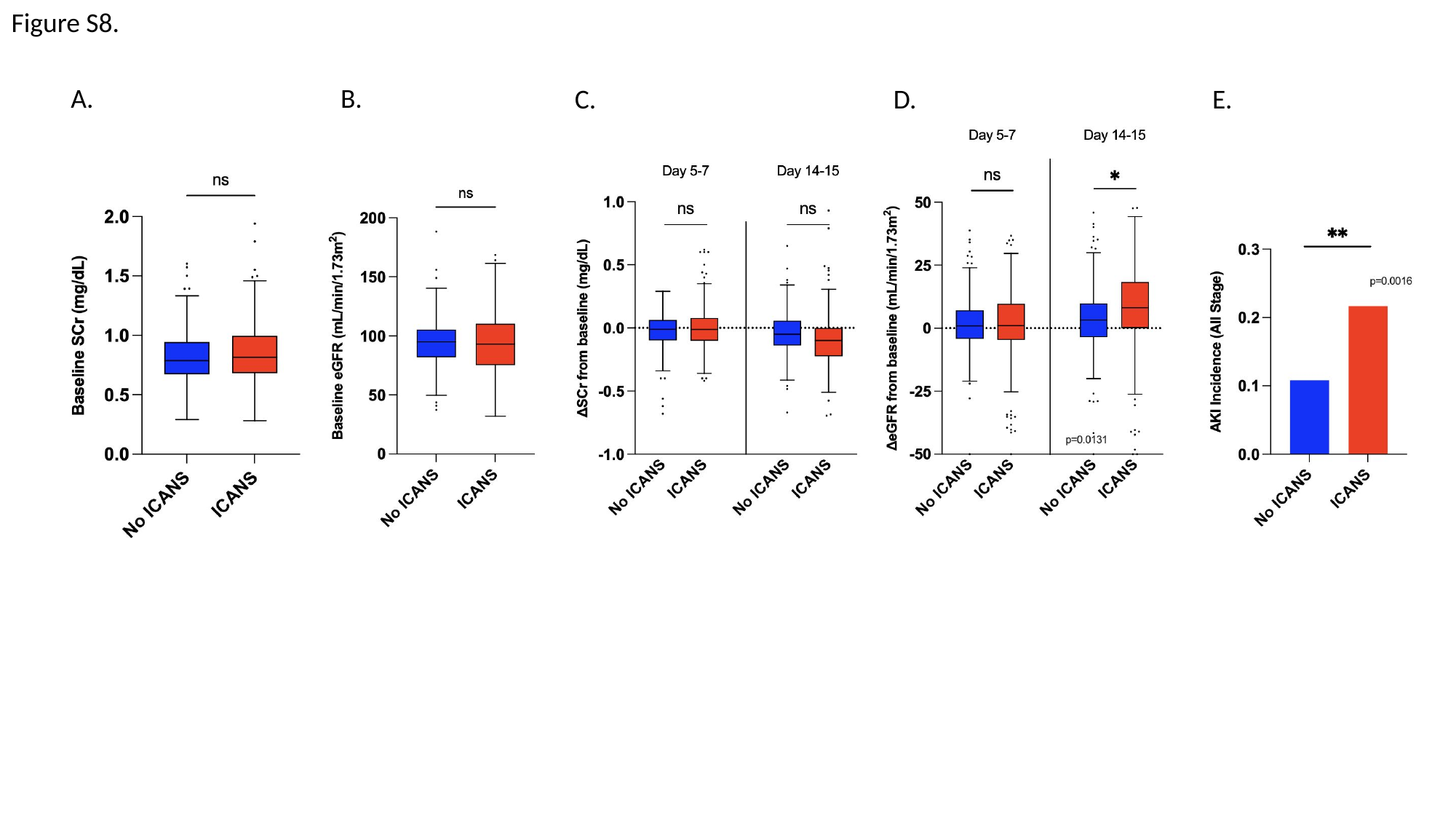

Figure S8.
A.
B.
C.
D.
E.

Supplement: Figure S8 — Baseline and change in kidney function following CAR T therapy grouped by patient ICANS status. [file crc-24-0250_figure_s8_suppsf8.pptx]
