# Supplementary material for: Hypophosphatemia Correction Reduces ICANS Incidence and Duration in CAR T-cell Therapy: A Pooled Clinical Trial Analysis
Source: Cancer Res Commun. 2024 Oct 3;4(10):2589–97. doi: 10.1158/2767-9764.CRC-24-0250 (PMC11448391; doi:10.1158/2767-9764.CRC-24-0250)
Supplement: Supplemental Figure Legends [file crc-24-0250_supplemental_figure_legends_suppsfl.docx]

**Supplemental Figure Legends**

**Supplemental Figure 1. Nadir serum electrolyte concentrations grouped by patient ICANS status.**

Solid bars represent median nadir electrolyte concentration +/-SEM and dotted horizontal line represents thresholds for (A) hypophosphatemia (2.5mg/dL), (B) hypokalemia (3mEq/L), (C) hypomagnesemia (1.3mg/dL), and (D) hypocalcemia (8mg/dL), respectively. *, P<0.05, **, P<0.01; Mann-Whitney U test.

**Supplemental Figure 2. Trends in serum electrolyte concentrations following CAR T-cell infusion grouped by patient ICANS status.**

Trend of serum (A) phosphorus, (B) potassium, (C) magnesium, (D) calcium following time of CAR T-cell infusion, stratified by patient ICANS status, represented as median (solid line) with upper and lower bonds of the 95% confidence intervals (shaded area).

**Supplemental Figure 3.** **ICANS incidence and time to symptom onset.**

(A) Bar graph showing number of patients with ICANS grouped by stage. (B) Time to ICANS symptom onset measured in days following CAR T infusion represented as a histogram with overlying gaussian curve.

**Supplemental Figure 4. Association of hypophosphatemia incidence by ICANS grade.** Patients with hypophosphatemia stratified by maximum ICANS grade documented post-CAR-T cell infusion. Association of greater hypophosphatemia incidence was statisticaly significant (p<0.01, one-way ANOVA).

**Supplemental Figure 5. Associations between pre-infusion tumor burden and ICANS and hypophosphatemia.** (A, B) Baseline pre-infusion tumor burden of patients with NHL (cm^2^) shows no association with incidence of ICANS or hypophosphatemia. (C,D) Baseline pre-infusion tumor burden of patients with ALL (peripheral blast:leukocyte ratio) is associated with decreased incidence of ICANS, and is weakly associated with decreased incidence of hypophosphatemia. Bar graphs represent means of both groups, +/-SEM, and were statistically compared using the unpaired t-test.

**Supplemental Figure 6. Histogram plots of nadir phosphorus values stratified by ICANS status.**

**Supplemental Figure 7. Coincidence of serum hypocalcemia with hypophosphatemia.**

Pie chart showing proportion of patients with serum hypocalcemia (n=49) who had concurrent serum hypophosphatemia (n=43).

**Supplemental Figure 8.** **Baseline and change in kidney function following CAR T therapy grouped by patient ICANS status.**

(A) Baseline serum creatinine (Scr) and (B) baseline estimated glomerular filtration rate (eGFR) in CAR T-cell patients stratified by patient ICANS status. (C) Change in serum creatinine from baseline and (D) change in eGFR from baseline on day +5-7 and day +14-15 post-infusion stratified by patient ICANS status. Each patient’s greatest change was plotted if multiple values were available for each time range. Data are represented as Tukey box and whisker plots, where the box represents median and interquartile range (IQR) and whiskers extend to the 75th percentile plus 1.5 times the IQR on the top and 25th percentile minus 1.5 times the IQR on the bottom. E) Bar graph depicting AKI incidence in CAR T-cell recipients stratified by ICANS status. *p<0.05, **p<0.01; Mann-Whitney U test.
